# Transcranial Acoustic Metamaterial Parameters Inverse Designed by Neural Networks

**DOI:** 10.34133/bmef.0030

**Published:** 2023-09-25

**Authors:** Yuming Yang, Dong Jiang, Qiongwen Zhang, Xiaoxia Le, Tao Chen, Huilong Duan, Yinfei Zheng

**Affiliations:** ^1^ College of Biomedical Engineering and Instrument Science, Zhejiang University, Hangzhou, Zhejiang 310027, China.; ^2^ Key Laboratory of Marine Materials and Related Technologies, Zhejiang Key Laboratory of Marine Materials and Protective Technologies, Ningbo Institute of Material Technology and Engineering, Chinese Academy of Sciences, Ningbo 315201, China.

## Abstract

*Objective:* The objective of this work is to investigate the mapping relationship between transcranial ultrasound image quality and transcranial acoustic metamaterial parameters using inverse design methods. *Impact Statement:* Our study provides insights into inverse design methods and opens the route to guide the preparation of transcranial acoustic metamaterials. *Introduction:* The development of acoustic metamaterials has enabled the exploration of cranial ultrasound, and it has been found that the influence of the skull distortion layer on acoustic waves can be effectively eliminated by adjusting the parameters of the acoustic metamaterial. However, the interaction mechanism between transcranial ultrasound images and transcranial acoustic metamaterial parameters is unknown. *Methods:* In this study, 1,456 transcranial ultrasound image datasets were used to explore the mapping relationship between the quality of transcranial ultrasound images and the parameters of transcranial acoustic metamaterials. *Results:* The multioutput parameter prediction model of transcranial metamaterials based on deep back-propagation neural network was built, and metamaterial parameters under transcranial image evaluation indices are predicted using the prediction model. *Conclusion:* This inverse big data design approach paves the way for guiding the preparation of transcranial metamaterials.

## Introduction

Manipulation and design of acoustic and electromagnetic metamaterials are the study of controlling waves by designing delicate structures. Some exotic acoustic properties can be obtained in acoustic metamaterials (AMMs) through ordered modulation of sound waves, such as negative reactance [1,2], acoustic cloak [3], acoustic focusing [4,5], abnormal Doppler characteristics [6,7], and canceling out the distortion layer such as skull [8–12]. Some scholars have found that by adjusting the parameters of the AMM, the influence of the skull distortion layer on the acoustic wave can be effectively eliminated [8,11]. By adding microstructure AMM before the distorted layer, the transmitted acoustic energy is substantially enhanced, and the reflected acoustic energy is reduced considerably. From a macroscopic point of view, the transcranial AMM “offsets” the distorted layer in the acoustics. While ultrasound as a biologically beneficial mechanical waveform has demonstrated practical implications in the biomedical field [13,14], the transcranial AMM used in this application has a characteristic response to ultrasound stimulation. Therefore, it is theoretically feasible for transcranial AMM to compensate for the attenuation and distortion effect of the skull.

However, designing a matching AMM structure for a specific acoustic effect requires designers to repeatedly perform complex numerical calculations on the structure [15]. This process consumes tremendous time and computing resources owing to its inherent iterative nature and requirement for full-field numerical simulation [16]. As the design becomes more complex and the changes in the structure become more diverse, problems will require more time and resources to solve. Moreover, owing to the incomplete theoretical model of transcranial AMM, the interaction mechanism between transcranial imaging and AMM is unclear. It is challenging to design the structure of transcranial AMM with incomplete numerical models only by numerical calculations. Therefore, it is urgent to find new ways to simplify or even replace traditional design methods [17].

The advancement in the field of neural networks has shed light on developing new methods to overcome the challenges mentioned above. Material design incorporates structure as an additional design variable and thus offers new routes for the development of materials with desired properties. It then allows for top-down reverse design approaches that map the required system performance onto materials structure and properties [18]. Currently, neural networks driven by big data have been used in the design and optimization of metamaterial parameters and achieved remarkable results [19–22]. In AMM research, neural-network-based approaches have been proposed for the inverse design of photonic structures [22], acoustic cloak structures [23], acoustic absorber structures [24,25], and so on. The inverse design process allows fast and accurate prediction of the metamaterial structure and properties with neural networks.

**Fig. 1.**
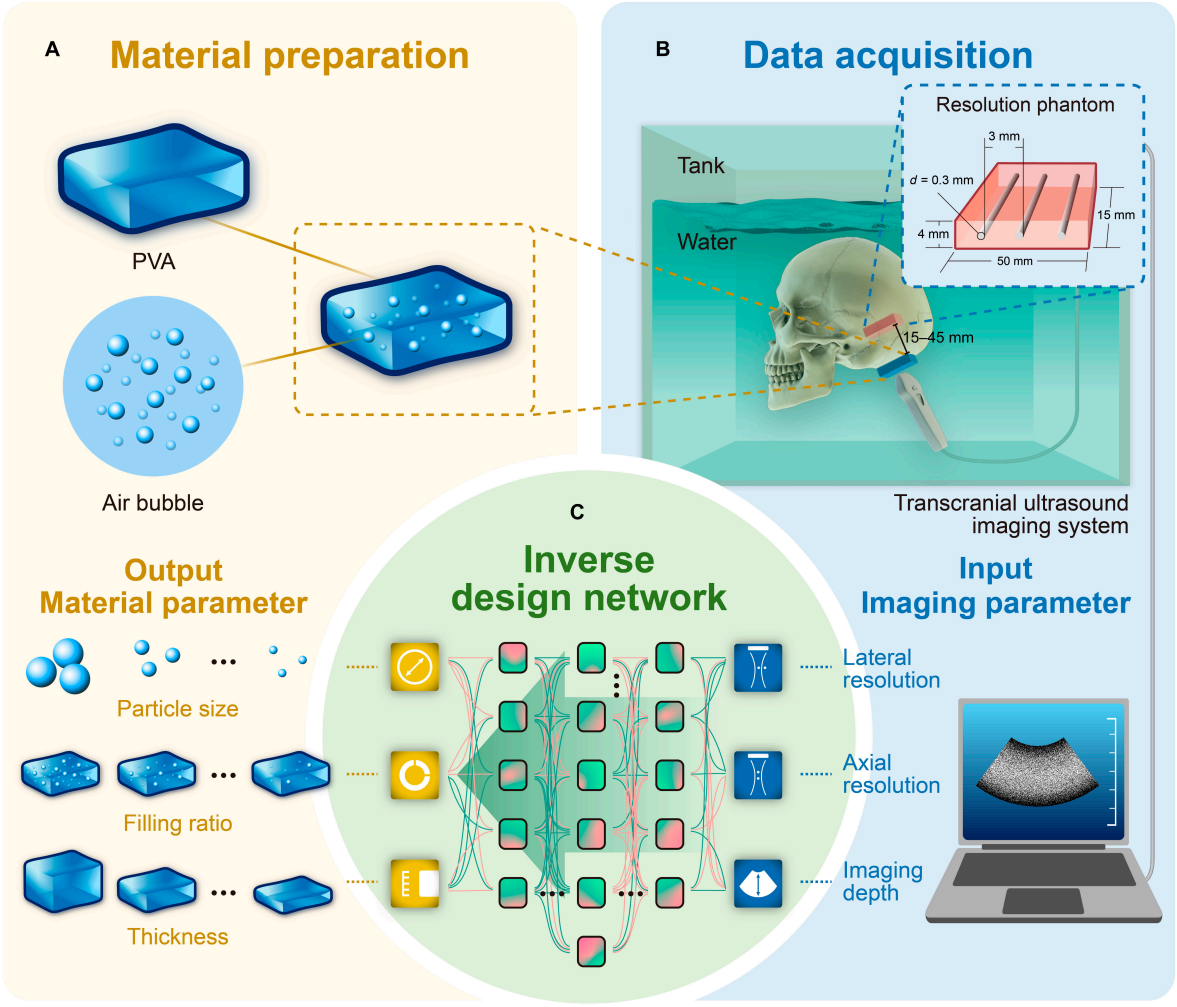
Neural-network-decision-based parameter prediction method for transcranial AMM. The inverse model inputs imaging parameters and predicts the corresponding material parameters. (A) The transcranial AMM preparation and the main parameter of the transcranial AMM. (B) The imaging parameter data acquisition from the transcranial ultrasound imaging system. (C) The reverse design approaches map the required transcranial ultrasound imaging system performance onto AMM parameters.

This study proposes a neural-network-decision-based parameters prediction method for transcranial AMM shown in Fig. 1. The reverse design approaches map the required transcranial ultrasound imaging system performance onto AMM parameters. The big data model was used to find the mapping relationship between the parameters of transcranial AMM (particle size, filling ratio, and thickness) and the quality of the transcranial image (lateral resolution, axial resolution, and imaging depth). Then, material parameters were deduced from the imaging experiment to guide AMM preparation. Notably, this framework allows us to design transcranial AMM using realistic transcranial ultrasound imaging results, which is novel for metamaterial design techniques.

## Results

### Designing of transcranial AMM parameter prediction neural network

**Fig. 2.**
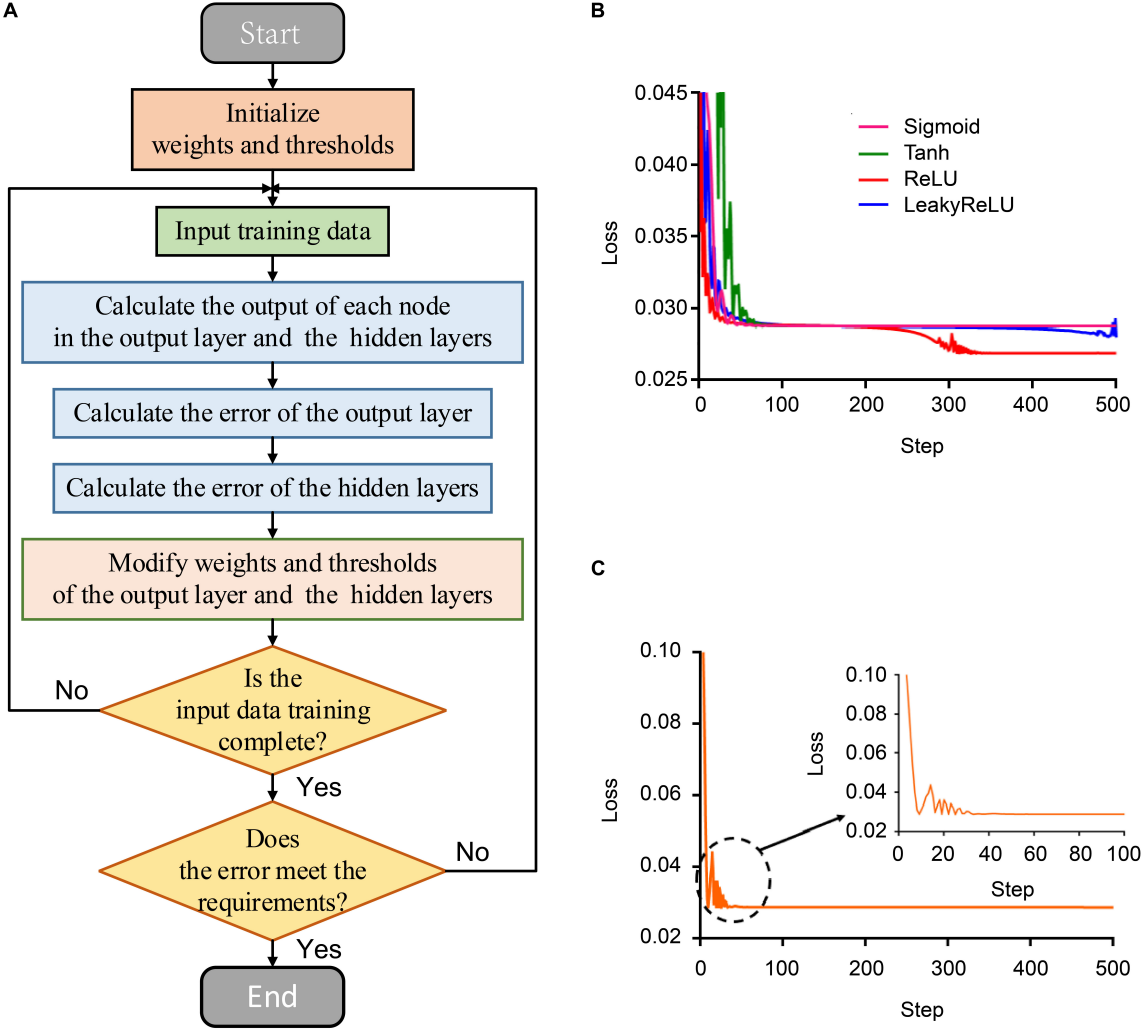
Results for the transcranial AMM parameters prediction neural network. (A) The chart flow of the BP algorithm. (B) Variation diagram of loss in the training process of different activation functions. (C) Variation diagram of loss in the training process of the BP neural network.

In this study, we use a back-propagation (BP) neural network to find the mapping relationship between the parameters of transcranial AMM and the quality of the transcranial ultrasound image. The BP neural network is one of the most widely used neural network models, suitable for finding the mapping or matching relations between data [26]. There is no relationship in time or space between the data in the dataset we used, and the dataset has few dimensions. Therefore, we choose a BP neural network to predict the parameters of transcranial AMM. The flow chart of the BP neural network is presented in Fig. 2A.

We took the imaging depth, lateral resolution, and axial resolution as the input of the prediction model. For the output, we took the average particle size, filling ratio, and thickness of the AMM. We determined that the number of nodes in the input layer should be 3, and the number of nodes in the output layer should also be 3. In this paper, we chose the Adam (adaptive moment estimation) [27] optimizer and ReLU (rectified linear units) [28] activation function, as shown in Fig. 2B. The other hyperparameters were determined by experiments. The mean square error (MSE) and mean absolute error (MAE) were used to evaluate the reliability of the model, while hyperparameters were adjusted. The following equations are used to express MSE and MAE:

$$\text{MSE}=\frac{1}{N}\sum_{i=1}^{N}{\left(Y_{i}-y_{i}\right)}^{2}$$ (1)

$$\text{MAE}=\frac{1}{N}\sum_{i=1}^{N}\left|Y_{i}-y_{i}\right|$$ (2)

where *Y_i_* represents the expected output of the model, *y_i_* represents the actual output of the model, and *N* represents the number of samples.

**Fig. 3.**
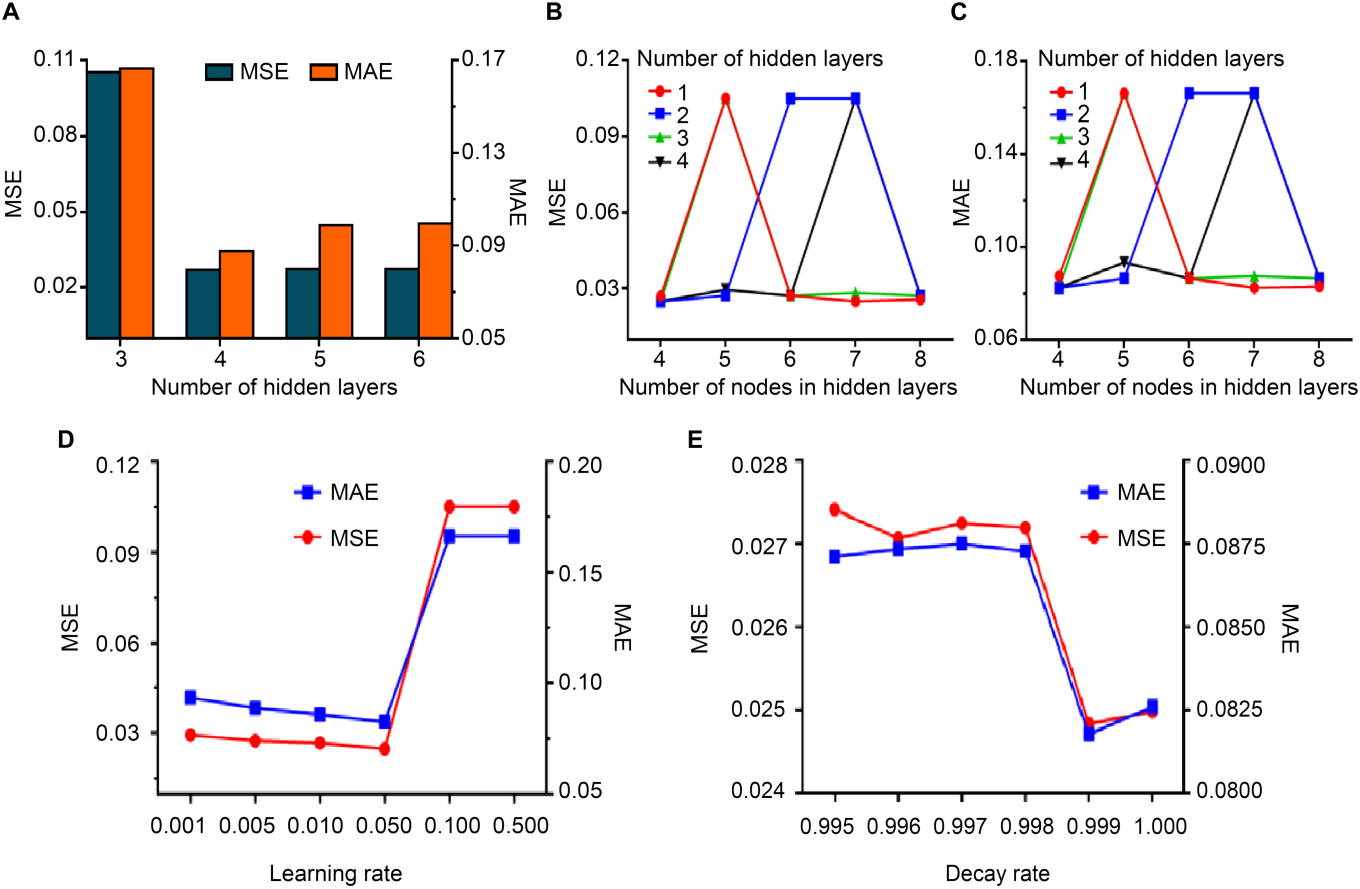
Experimental results of hyperparameters comparison. (A) MSE and MAE of the different number of hidden layers. (B) MSE of the different number of nodes in different hidden layers. (C) MSE of the different number of nodes in different hidden layers. (D) MSE and MAE of different learning rates. (E) MSE and MAE of different decay rates.

To determine the number of hidden layers, we fixed the remaining hyperparameters and set the number of hidden layers to 3, 4, 5, and 6. The learning rate is set to 0.05, ReLU function is used as the network activation function, Adam algorithm is selected as network optimization function, and the number of nodes in each hidden layer is fixed at 4. After 500 iterations, the number of hidden layers was fixed at 4, which minimizes prediction error. The experimental results are shown in Fig. 3A.

To determine the number of nodes in each hidden layer, we first changed the number of nodes in the first hidden layer (4, 5, 6, 7, and 8) while fixing the number of nodes in the following hidden layer to 4 and then chose the number of nodes that produced the optimal results. The optimal nodes of the first hidden layer were fixed, and the number of nodes in other hidden layers was determined similarly. Each experiment was iterated 500 times. The experimental results of the first layer are shown in Fig. 3B and C. According to the experimental results of the prediction model, the number of nodes in hidden layers was set to 7-4-4-4, and the overall network structure was 3-7-4-4-4-3.

The fixed learning rate method cannot effectively achieve fast network convergence with a small dataset, so we chose the exponential decay learning rate method to dynamically and flexibly set the learning rate. The following equations are used to express the exponential decay learning rate:

$$\mathrm{Lr}={\mathrm{Lr}}_{\text{base}}\times \tau^{\frac{\text{global}\_\text{step}}{\text{decay}\_\text{steps}}}$$ (3)

where *Lr* represents the learning rate, *Lr*_base_ represents the base learning rate, *τ* represents the decay factor (taken from a constant between 0 and 1), global_step represents the current number of training steps, and decay_steps represents the decay rate (the decay rate is set to 1, meaning that the learning rate is updated every iteration). We implemented several common choices of learning rate; the experimental results are shown in Fig. 3D. According to the experimental results, the basic learning rate was set to the optimal value (0.05). Then, we experimented to determine the decay factor of the learning rate; the experimental results are shown in Fig. 3E. According to the experimental results, the decay factor of the learning rate was set to 0.999.

On the basis of the experiments, the hyperparameters of the BP neural network used in this paper are shown in Table S1. Briefly, the optimization algorithm is Adam method, the activation function is ReLU function, the number of hidden layers is 4, the number of hidden layer nodes is 7-4-4-4, the learning rate is 0.05, the learning rate attenuation factor is 0.999, and the dropout probability value is 0.97.

### The preparation of hydrogel-based transcranial AMM

**Fig. 4.**
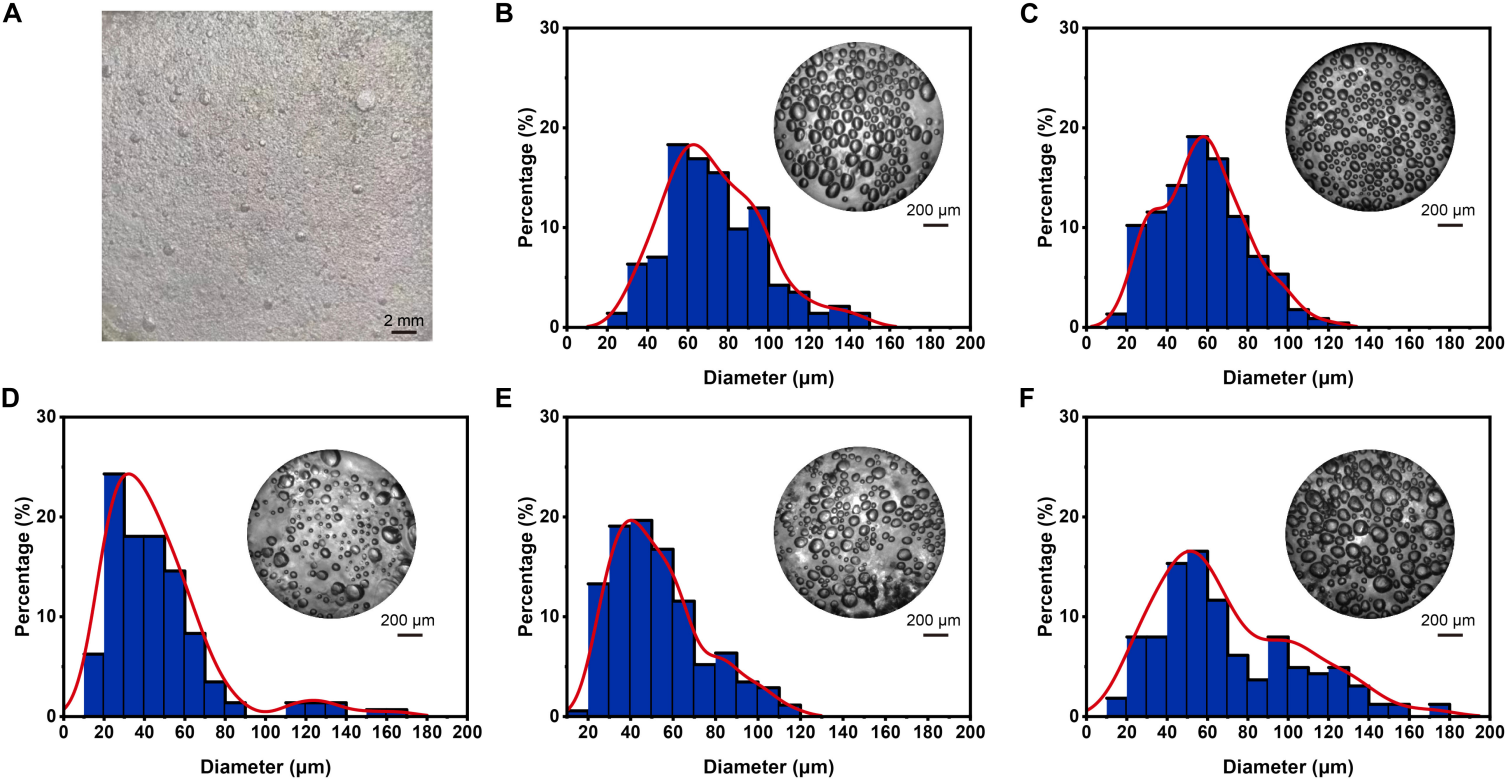
Characterizations of transcranial AMM. (A) A sample of transcranial AMM with 2 mm in thickness. Scale bar, 2 mm. Hydrogel-based metamaterials with adjustable bubble apertures, which were achieved by adding different amounts of PAAm [e.g., (B) 0.1 wt%, (C) 0.5 wt%, (D) 1 wt%, (E) 2 wt%, and (F) 3 wt%]. Scale bars, 200 μm.

A series of hydrogel-based metamaterials with adjustable bubble apertures were fabricated via a 2-step method. First, hydrophilic monomer acrylamide (AAm) was polymerized at room temperature in the presence of *N*,*N*′-methylene bis(acrylamide) as a cross-linker, ammonium persulfate as an initiator, and *N*,*N*,*N*′,*N*′-tetramethylethylenediamine as an accelerator. After reaction for 24 h, the prepared polyacrylamide (PAAm) hydrogels were crushed and swollen in the water to remove unreacted monomers. Then, the fully swollen PAAm hydrogel fragments were freeze-dried and refined into smaller powders. Through the freeze-thawing method, the final hydrogel-based metamaterials were obtained by doping different amounts of the above powder into poly(vinyl alcohol) (PVA) solution (10 wt%) with the assistance of different stirring rates and sealed in a sandwiched mode. By adding different amounts of PAAm, the bubble apertures can be adjusted. Transcranial AMM with different amounts of PAAm is characterized in Fig. 4.

### Training of transcranial AMM parameter prediction neural network

**Fig. 5.**
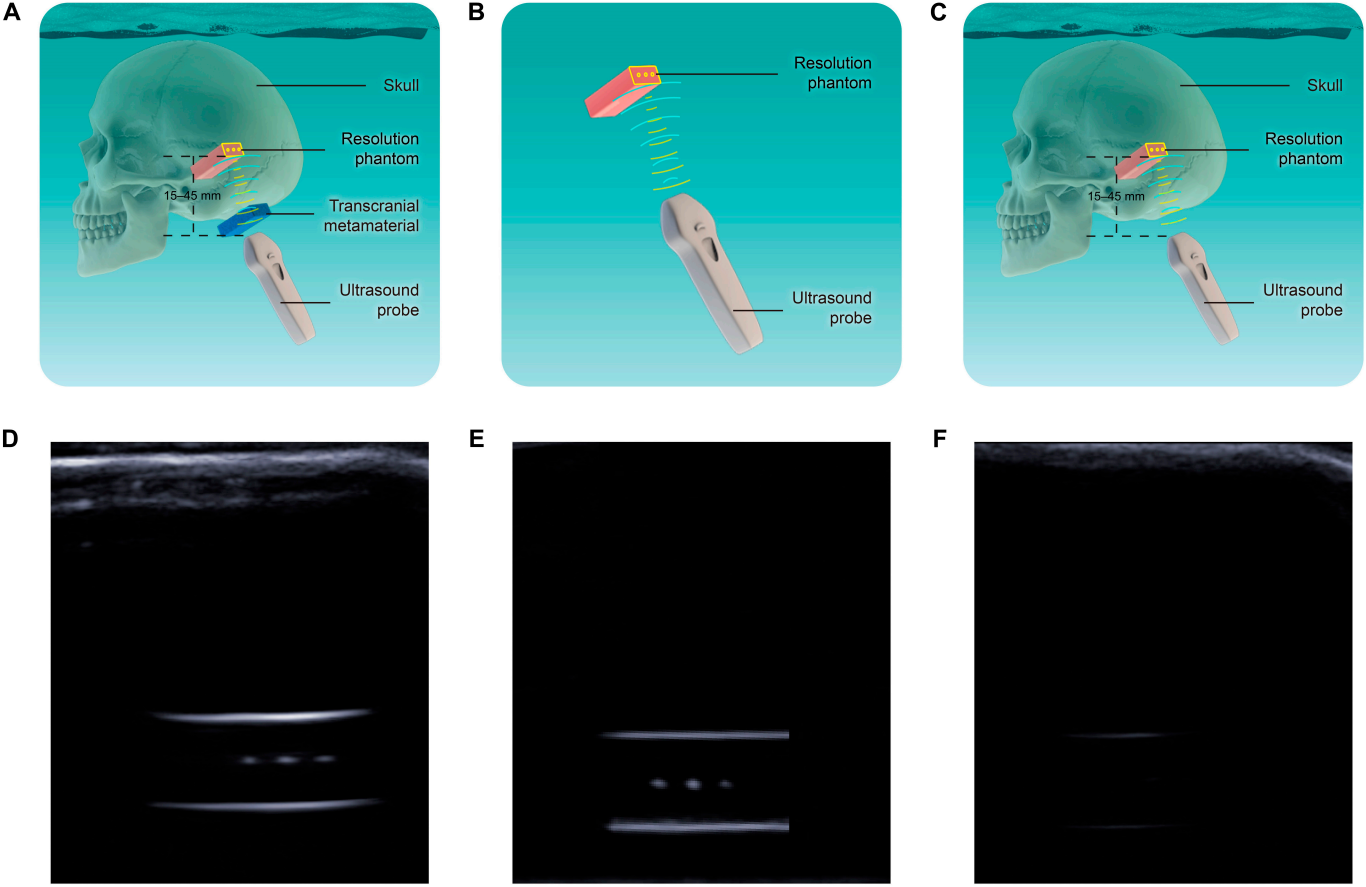
Schematic diagram and renderings of the transcranial ultrasound imaging experiment. (A) Transcranial ultrasound imaging with the presence of transcranial AMM. (B) Direct imaging with the 3-mm resolution phantom. (C) Transcranial ultrasound imaging without the presence of transcranial AMM. (D to F) Corresponding ultrasonic images of (A), (B), and (C), respectively.

Data were collected through the transcranial ultrasound imaging experimental system, as shown in Fig. 1B. The experimental system was mainly composed of 5 parts: ultrasonic imaging system, transcranial AMM, skull, water tank, and resolution phantom [29]. The ultrasonic imaging system was used to collect the transcranial images. Transcranial AMM was used to enhance the energy of ultrasonic penetration through the skull. The resolution phantom was the imaging target of the system. A resolution phantom for ultrasonic craniotomy was designed and manufactured to measure imaging resolution parameters. Figure 1 shows the schematic diagram and the picture of the phantom with a resolution of 3 mm. Transcranial ultrasound imaging was carried out using the prepared transcranial AMM. Figure 5 shows the schematic diagram and effect of the transcranial ultrasound imaging experiment. In Fig. 5A and C, the ultrasound probe penetrated the human skull to image the target phantom with and without transcranial AMM. Figure 5D and F shows ultrasound images obtained from Fig. 5A and C, respectively. Figure 5B and E is the control group in water without the skull. As shown in Fig. 5F, we can see that without the transcranial AMM, the target phantom cannot be distinguished. However, with the transcranial AMM in Fig. 5D, the targets phantom can be clearly imaged. In Fig. 5D and E, the horizontal hyperechoic lines represent the echo signal from the upper and lower edges of the resolution phantom, and the hyperechoic dots represent the cross-sectional imaging of the wire wrapped in the resolution phantom, which means that the targets can be clearly distinguished. In contrast to Fig. 5F, in which the presence of the cranium results in attenuation of the signal, only the blurry top and bottom edges of the mold can be seen, and the internal information from the thin metal lines is absent.

The main parameters (mean particle size, filling ratio, and thickness) were changed to prepare the transcranial AMM with different parameter ratios. The average particle size ranges from 10 to 400 μm, the filling ratio ranges from 0.1% to 5%, and the material thickness varies from 2 to 8 mm. On the basis of the transcranial AMM, ultrasonic craniotomy images were collected. The evaluation indices of ultrasonic image quality (lateral resolution, axial resolution, and imaging depth) were obtained by offline calculation of the relevant image data. This prepared the data for the subsequent prediction of the parameters of the transcranial AMM by the neural network.

The ultrasonic signals sent by the ultrasonic probe are sequentially passing through the transcranial AMM and the skull to image the resolution phantom placed inside the skull. Ultrasonic images were collected at imaging depths of 15, 17.5, 20, 22.5, 25, 27.5, 30, 32.5, 35, 37.5, 40, 42.5, and 45 mm, and the corresponding lateral resolution and axial resolution resolutions were calculated offline. The full width at half maximum (FWHM) is a standard resolution evaluation method in the imaging field, which is usually obtained by calculating the point spread function of the system. This study adopted an energy drop of 8 dB as the standard FWHM to measure the resolution of ultrasonic images.

On the basis of the above design of the ultrasonic craniotomy experiment, a total of 1,456 sets of data were collected. The collected data were further preprocessed to ensure the effectiveness of network training. The first step in this process is data cleaning. Data cleaning principles are as follows: The image data items with poor effect are directly deleted. The offline calculation achieves the resolution using the average peak FWHM of the 3 lines inside the phantom. The FWHM of the central peak is 0.5 times away from the average value, indicating that this value is abnormally higher or lower than the 2 values before and after it, and this group of data is discarded from consideration. After data cleaning, 915 sets of available compelling data were screened out and split into 3 distinct groups: 60% of the data were for training, 20% was for validation, and 20% was for final testing. The adequate data were normalized for deviation. By scaling the data according to the scale, the sample data fell in a range between 0 and 1.

As shown in Fig. 2C, the loss values of the BP neural network for the test datasets can converge to a low-value region after around 100 iterations. Regarding error evaluation indices, the MSE and MAE of the BP neural network the test datasets are 0.02479 and 0.08195, respectively. Furthermore, the training time is 2.11396 s. Briefly, the BP neural network can obtain relatively good prediction results after the iterative training once the hyperparameters are set to the optimal value.

The BP network model with high prediction accuracy was further used to predict transcranial AMM parameters in the region with a rich cerebrovascular distribution (20 to 40 mm in depth). The lateral and axial resolutions were fixed at 2 mm, and the predicted results are shown in Table S2. It can be seen that the network model built in this study can establish the mapping and matching relationship between the quality of transcranial images and the parameters of AMM and then guide the actual preparation of transcranial AMM.

**Fig. 6.**
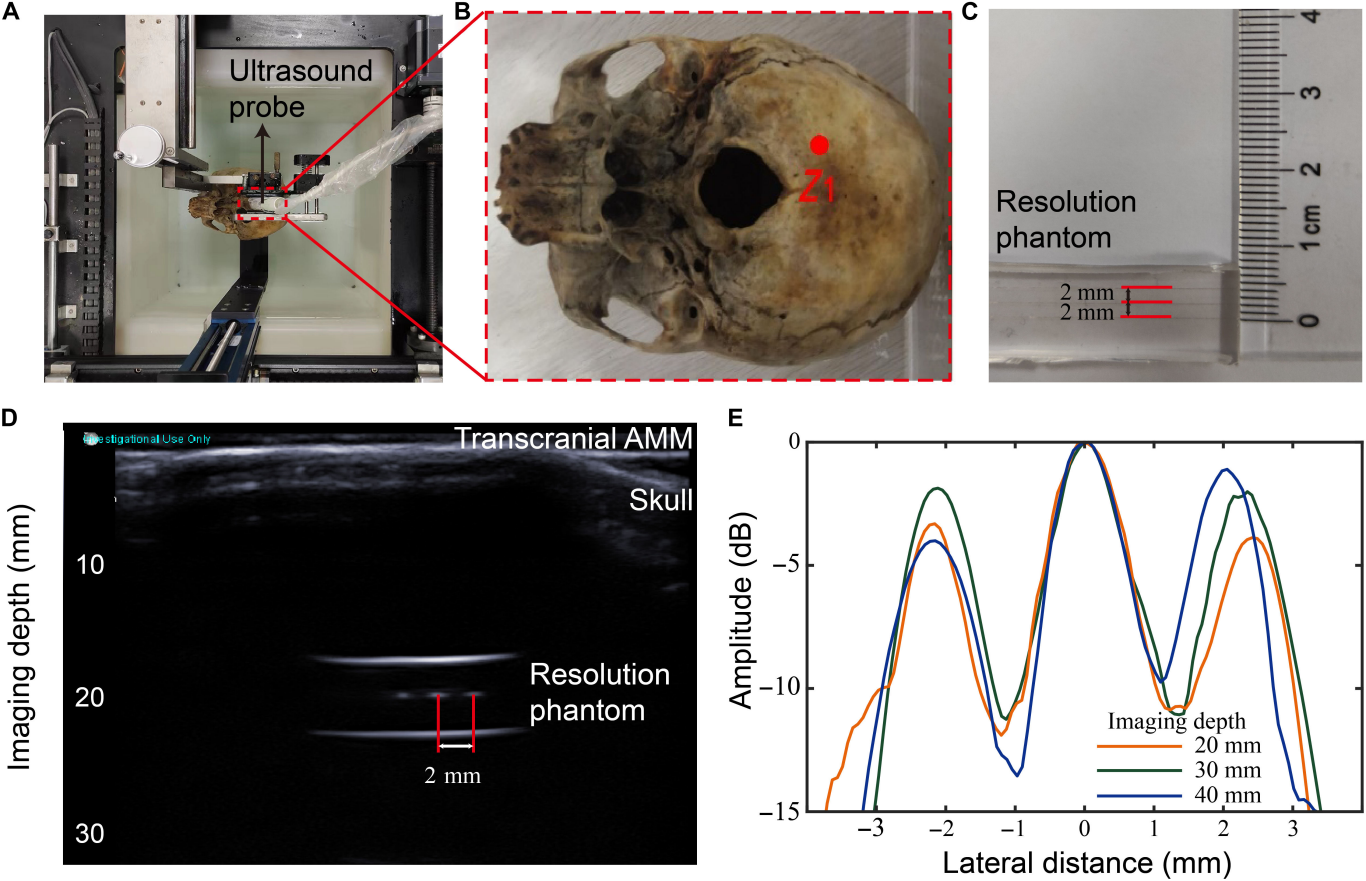
Lateral profile of energy distribution for different transcranial AMMs at different imaging depths. (A) Transcranial ultrasound imaging experiment device. (B) Data acquisition through the cerebellum fossa (*Z*_1_). (C) The photo of the resolution phantom. (D) Transcranial ultrasound image of 2-mm resolution phantom. (E) Lateral energy distribution profile at different imaging depths for different transcranial AMMs.

After using the BP network model to predict transcranial AMM parameters for 2 mm resolution at different imaging depths, we prepared different transcranial AMMs to verify their ultrasound imaging qualities. Data were collected through the transcranial ultrasound imaging experimental system, as shown in Fig. 6A. The ultrasound probe was used to collect the transcranial images through the cerebellum fossa (Fig. 6B). Through computed tomography scan (Philips/iCT 256), the thickness of skull at *Z*_1_ is about 1.53 mm. Transcranial AMM was used to enhance the energy of ultrasonic penetration through the skull. The 2-mm resolution phantom was the imaging target of the system (Fig. 6C). It can be seen from Fig. 6E that the signal amplitude of the 3 wires in the resolution phantom is not uniform because of the nonuniformity of the skull. At 20-, 30-, and 40-mm imaging depth, different transcranial AMMs can distinguish the 2-mm resolution phantom. The quality of transcranial images was further verified through the actual preparation of transcranial AMM, and the practicality of the network model was demonstrated.

## Conclusions

The skull’s strong attenuation and distortion effect on ultrasound make it challenging to realize transcranial ultrasound imaging. It is of great theoretical and clinical importance to establish the mapping relationship between the quality of transcranial ultrasound images and the parameters of transcranial AMM. In this study, image data were collected using a skull body model and the resolution phantom, and the dataset was constructed. Prediction models based on deep BP neural networks were designed, and the actual prediction of the parameters of a metamaterial was carried out, which is of great importance for guiding the preparation of transcranial AMM. However, this study still has some limitations, and improvements can be made in the following ways: (a) by further improving the quality of transcranial ultrasound image acquisition; (b) by further expanding the scale of neural network datasets (such as increasing the number of parameters of the transcranial AMM); (c) by further optimizing the model and improving the accuracy of predicting the parameters of transcranial AMM (for example, including the introduction of a physics guided inverse design model [30]) or the use of multilayer perceptron network to mine the complex relation between the transcranial AMM parameters and the imaging quality characteristics [31]; (d) by improving the preparation method of the transcranial AMM (for example, through microfluidic technology [32,33]) and improving the preparation of the transcranial AMM predicted by the network model to verify the accuracy of the model prediction.

## Materials and Methods

### Experiment system

The experimental system was mainly composed of 5 parts: ultrasonic imaging system, transcranial AMM, skull, water tank, and resolution phantom. The ultrasonic imaging system was used to collect the transcranial images. Transcranial AMM was used to enhance the energy of ultrasonic penetration through the skull. The resolution phantom was the imaging target of the system. The resolution phantom was made of polydimethylsiloxane wrapped in 3 fine metal wires, and the spacing of the metal wires was the measurement index of resolution. The diameter of the gold filament was 0.3 mm, and the length, width, and thickness of the separation rate die were 50, 15, and 4 mm, respectively.

In this study, a SonixTouch (Ultrasonix, Richmond, Canada) ultrasonic imaging system was used to control the L14-5 linear array transducer (plane size of 45 mm × 10 mm) with a transmission center frequency of 5-MHz ultrasonic signals. A SonixDAQ (Ultrasonix, Richmond, Canada) multichannel echo data acquisition module was used to collect ultrasonic echo signals, obtain images at different imaging depths, and conduct offline processing on the echo signals to calculate image resolution, contrast, and contrast noise ratio.

## Data Availability

The data are available from the authors upon a reasonable request.

## Supplementary Materials

Supplementary 1 Table S1. Hyperparameters of BP neural network prediction model. Table S2. Prediction results of 2-mm resolution network at different imaging depths.
