# Supplementary material for: Transcranial Acoustic Metamaterial Parameters Inverse Designed by Neural Networks
Source: BME Front. 2023 Sep 25;4:0030. doi: 10.34133/bmef.0030 (PMC10521689; doi:10.34133/bmef.0030)
Supplement: Supplementary 1 — Table S1. Hyperparameters of BP neural network prediction model. Table S2. Prediction results of 2-mm resolution network at different imaging depths. [file bmef.0030.f1.docx]

**Table S1.** Hyperparameters of BP neural network prediction model.

| Hyperparameter | Value |
| --- | --- |
| Optimizer | Adam |
| Activation function | ReLU |
| Base learning rate | 0.05 |
| Decay factor of the learning rate | 0.999 |
| Number of nodes in the input layer | 3 |
| Number of nodes in the output layer | 3 |
| Number of hidden layers | 4 |
| Number of nodes in hidden layers | 7-4-4-4 |

**Table S2.** Prediction results of 2 mm resolution network at different imaging depths

| Imaging quality | | Prediction transcranial AMM parameters | | |
| --- | --- | --- | --- | --- |
| Imaging depth (mm) | Resolution (mm) | Diameter (μm) | Filling ratio (%) | Thickness (mm) |
| 20 | 2 | 276.48 | 1.98 | 4.86 |
| 30 | 2 | 251.59 | 2.07 | 5.01 |
| 40 | 2 | 226.71 | 2.17 | 5.16 |
